# What Does Not Work in Adolescent Sexual and Reproductive Health: A Review of Evidence on Interventions Commonly Accepted as Best Practices

**DOI:** 10.9745/GHSP-D-15-00126

**Published:** 2015-08-31

**Authors:** Venkatraman Chandra-Mouli, Catherine Lane, Sylvia Wong

**Affiliations:** ^a^​ World Health Organization, Department of Reproductive Health and Research, Geneva, Switzerland; ^b^​ United States Agency for International Development, Bureau for Global Health, Office of Population and Reproductive Health, Washington DC, USA; ^c^​ United Nations Population Fund, New York, NY, USA

## Abstract

Youth centers, peer education, and one-off public meetings have generally been ineffective in facilitating young people’s access to sexual and reproductive health (SRH) services, changing their behaviors, or influencing social norms around adolescent SRH. Approaches that have been found to be effective when well implemented, such as comprehensive sexuality education and youth-friendly services, have tended to flounder as they have considerable implementation requirements that are seldom met. For adolescent SRH programs to be effective, we need substantial effort through coordinated and complementary approaches. Unproductive approaches should be abandoned, proven approaches should be implemented with adequate fidelity to those factors that ensure effectiveness, and new approaches should be explored, to include greater attention to prevention science, engagement of the private sector, and expanding access to a wider range of contraceptive methods that respond to adolescents’ needs.

## INTRODUCTION

The 1994 International Conference on Population and Development (ICPD) was a landmark event for adolescent sexual and reproductive health (ASRH). Thanks to the efforts of advocates from around the world, the pressing need to address the sexual and reproductive health (SRH) of young people including adolescents was acknowledged in the ICPD’s Programme of Action.^1^

We submit that in 1994, while there was some awareness and understanding of the SRH needs and concerns of (mostly unmarried) adolescents in low- and middle-income countries (e.g., high rates of early and unintended pregnancy, early childbearing, unsafe abortion, and sexually transmitted infections), there was limited understanding of effective ways of responding to these needs and problems.^2^ The knowledge available at that time suggested that adolescents and young people lacked understanding of sexuality, reproduction, and sexual and reproductive health; that they were not getting the information and education they needed at home, at school, or elsewhere in their communities; and that they were neither able nor willing to obtain health services because they were not “youth friendly.” The social environment was not conducive to acknowledging adolescent sexuality or their right to healthy sexual development. Laws and policies around providing information and services to unmarried adolescents were generally restrictive, and even where supportive laws and policies existed, these were contradicted by others or not enforced.

There was little evidence based on research or practical experience on effective ways of providing young people with information, education, and health services, or equipping them with skills to protect them from risks. Further, there was limited understanding of approaches to address deeply held community norms and biases against premarital sexual activity among adolescents.

Moreover, existing reproductive health programs were primarily targeted to adult women. Married adolescents fell into this category, as marriage usually conferred adult status and thus the right to access services. Yet while it was acceptable for married adolescents to access these services when compared with their unmarried peers, the quality of the services they received was wanting, as they were not sensitive to the age-specific and developmental needs of young women who might want to delay their first pregnancy or who were pregnant for the first time.

Much has happened over the last 20 years. Interventions that acknowledge the rights of adolescents and respond to their needs have been designed and piloted; policies and strategies formulated; projects and programs implemented; and research studies and evaluations conducted. Although there are still many gaps in our knowledge and understanding, we have a much better picture of the needs and problems of adolescents in low- and middle-income countries.^3^^,^^4^ We also have a better understanding of what works—and what does not—in responding to their needs and problems.

A number of recent publications have pointed to interventions and intervention-delivery mechanisms that have been shown to improve adolescent sexual and reproductive health.^5^^-^^7^ They also argue that some of the interventions and intervention-delivery mechanisms have been shown to be ineffective. Despite this evidence, ineffective interventions and ineffective ways of delivering them continue to be widespread, and interventions that have been shown to be effective are often delivered ineffectively. As a result, human and financial resources are invested without any positive outcomes, and questions are raised about the value of investing in ASRH policies and programs, especially at scale.

Although there are gaps in our understanding of adolescent needs and problems in low- and middle-income countries, a growing body of evidence points to interventions that work and ones that do not.

Our review suggests at least 5 thematic areas that challenge our ability to demonstrate significantly positive results in ASRH programming:

1. Significant numbers of adolescents are not adequately reached by the interventions intended for them.
2. Interventions that have been shown to be ineffective continue to be implemented.
3. Interventions that have been shown to be effective are delivered ineffectively.
4. Interventions have limited effects because they are delivered piecemeal.
5. Interventions are delivered with inadequate dosage (i.e., they are of low intensity or for a short duration) resulting in limited or transient effects.

### Adolescents Are Not Adequately Reached by Interventions Intended for Them

For an intervention to have an effect on adolescents’ knowledge, attitudes, beliefs, and behavior, it must first be able to reach them. Studies suggest that many adolescents, especially those with the most pressing SRH needs or who are most marginalized or vulnerable, are not being reached by interventions as intended by program planners. Additionally, many first-generation adolescent health programs implemented broad-based interventions intended to reach all adolescents in a given community or catchment area, rather than identifying and targeting those most vulnerable to health and social problems.

Many adolescents, especially those who are most marginalized or vulnerable, are not being reached by adolescent health programs.

While it is unrealistic to expect that 100% of all adolescents in a given setting will be reached by an intervention, a study by Erulkar et al.^8^ is illustrative of the limited reach of many programs against what is intended. The study showed that in a peri-urban setting on the outskirts of Addis Ababa, Ethiopia, only 1 in 5 boys aged 10–19 and less than 1 in 10 girls of the same age made a visit to a local youth center over a period of 1 year. During the same period, just over 1 in 4 boys and less than 2 in 10 girls were contacted by a peer educator from projects operating in the area. For boys and girls in the 10–14 years age group, the visit and contact rates were substantially less.

Similar results were found in other countries and settings; without dedicated outreach for specific subgroups of vulnerable adolescents (such as very young adolescents or married adolescents), the more advantaged (e.g., older and unmarried adolescents and youth) are more likely to be reached by traditional youth programs.^9^

A recent evaluation of the TARUNYA Project, which supported the Government of Jharkhand State, India, to implement its ASRH Program, found that community-education sessions conducted by trained community volunteers over a period of 5 years reached around 1 in 4 girls aged 10–14, compared with 1 in 3 girls aged 15–19.^10^ Among the 1,288 girls surveyed, the project was predominantly reaching older, unmarried and literate adolescent girls. Even in a part of the state where levels of community education were high, they reached only 1 in 5 boys aged 18–19, out of 210 boys surveyed.

### Popular Interventions Shown to Be Ineffective for Adolescents Continue to Be Implemented

Three widely implemented interventions—youth centers, peer education, and high-profile meetings on ASRH—have been shown to be ineffective in changing ASRH health knowledge, attitudes, beliefs, and behaviors, yet remain popular approaches among program implementers.

Youth centers, peer education, and high-profile meetings on ASRH have been shown to be ineffective yet remain popular approaches among implementers.

#### Youth Centers to Increase Uptake of Contraception and Other Health Services

Youth centers are usually conceptualized as meeting points and “one-stop shops,” which are intended to be a friendly, safe, and non-clinical environment where SRH information and services can be provided alongside other social services, such as recreational activities or Internet cafés. This has been and continues to be a very popular approach, beginning in the 1980s in Latin America. Yet a number of evaluations have shown this approach is not effective as it does not result in increased use of SRH services or in any meaningful SRH behavior change. A review of evaluations of 18 youth center programs from around the world found^11^:

- The youth centers were mainly used by a relatively small proportion of young people who lived nearby, mostly male.
- These young men were attending school or college, and were much older than the intended target age.
- The youth centers were mainly frequented for recreation purposes.
- There were no or very limited/transient effects on the use of SRH services or contraceptive methods.
- The cost per beneficiary was very high.

Results definitively show that despite being a popular strategy for ASRH programming, youth centers are not cost-effective for increasing uptake of SRH services among adolescents. It should be noted that while youth centers have not been shown to be effective in changing adolescent SRH behaviors, youth centers may provide other social benefits through the provision of recreational and other youth development programs and may promote socially desirable outcomes such as reductions in gang activity or the development of employable skills.

#### Peer Education to Encourage Safe Sexual Behavior

Peer education is widely applied to capitalize on the perceived social networks of adolescents, as well as being seen as an inexpensive and easy-to-implement intervention. Peer education is also believed to provide opportunities for repeat contact and to be more effective than adult-led approaches in reaching marginalized or vulnerable young people.

Five meta-analyses of peer education programs implemented in widely different contexts over many years have concluded that while these programs result in information sharing, on their own, they have very limited effects in promoting healthy behaviors and improving health outcomes among target groups.^12^^-^^16^ The meta-analyses also show that peer education programs mainly benefit peer educators (who are usually the recipients of training and supervision) rather than their intended beneficiaries.

Peer education programs mainly benefit peer educators rather than their intended beneficiaries.

Two recent studies reiterate the limited benefits of peer education. A study by Michielsen et al. in Rwanda reported that “time trends in sexual risk behavior … were not significantly different in students from intervention and control schools, nor was the peer education intervention associated with increased knowledge, perceived severity or perceived susceptibility.”^17^ Sexual risk behaviors comprised being sexually active, having sex in the last 6 months, and not using condoms at last sex. The study did find significantly reduced reported stigma with the peer education intervention. Similarly, an evaluation in South Africa found that even with intensive support to strengthen peer education programs (e.g., “developing an adult infrastructure of training and support, alongside a cohort of trained and supported peer educators”), positive changes were limited and piecemeal.^18^

Given this evidence, and the fact that peer education programs have been shown to contribute to information sharing, the authors agree with the suggestion by Michielsen et al. that “peer education might be more effective if it is integrated in holistic interventions and if the role of peer educators is redefined in a way that makes them more of a source of sensitization and referral to experts and services.”^17^

#### High-Profile Public Meetings to Inform Communities About Harmful SRH Practices and to Urge Them to Abandon These Practices

Bringing community members together to inform them about the risks of early marriage and female genital mutilation and urging them to abandon these practices—often in well-publicized one-off public sessions—has been shown to have little effect in changing these practices. Yet such activities continue to be conducted because they are relatively easier to organize and are more visible compared with approaches that initiate and sustain longer-term community conversations. Such longer-term approaches would include ongoing dialogue with community leaders and members to encourage them to critically examine their traditions and help them identify and address the factors that contribute to poor outcomes.^19^^-^^21^

### Interventions Shown to Be Effective Are Delivered With Inadequate Fidelity

For an intervention to have the desired effect on an adolescent’s knowledge, attitudes, practices, and behaviors (KAPB), it must be delivered effectively. Two interventions that have been shown to improve adolescents’ KAPB are providing them with comprehensive sexuality education and with appropriate SRH services. However, these interventions are often poorly implemented, lacking fidelity to the elements of the interventions that make them effective in the first place, or picking and choosing among a few approaches rather than implementing them together as a whole.

Comprehensive sexuality education and appropriate SRH services are proven practices but are often poorly implemented.

#### Providing Adolescents With Comprehensive Sexuality Education

Comprehensive sexuality education (CSE) has been well-evaluated and has been shown to improve adolescent SRH knowledge, attitudes, and behaviors when implemented well. In 2009, the United Nations Educational, Scientific and Cultural Organization (UNESCO) with other UN partners developed technical guidance on the development and implementation of quality CSE.^22^ Based on research by Kirby et al.,^23^ the document identified 18 characteristics of quality sexuality education programs that effectively increased knowledge, clarified values and attitudes, improved skills, and positively affected behavior. Twelve of these characteristics are related to the development, content, and delivery of sexuality education. These characteristics emphasize the importance of providing factual information and specifically addressing risky behaviors and strengthening protective factors. Participatory teaching methodologies are also essential so as to ensure the development of skills and self-efficacy to act on information.

More recently, Haberland conducted an analysis of evaluated CSE programs and noted that programs that incorporate an empowerment approach emphasizing gender and rights were particularly effective in improving reproductive health outcomes.^24^ Haberland’s analysis suggests that young people who have egalitarian attitudes about gender roles in their intimate relationships are more likely to delay sexual debut, use condoms, and practice contraception.

Studies show, however, that many school-based CSE programs are not implemented with adequate attention to these characteristics, and the curriculum content tends to be weak. This is illustrated by 3 key findings of a review, carried out by UNESCO and the United Nations Population Fund (UNFPA), of the CSE curricula in 10 countries of East and Southern Africa, which a region-wide initiative aims to address^25^:

1. Most curricula did not contain enough basic information about male/female condoms and contraception (including emergency contraception).
2. Key aspects of sex and sexual health were lacking, including information about reproduction, sexually transmitted infections, abortion, and where to access condoms and sexual health services.
3. Most curricula did not pay enough attention to empowering young people, building agency, or teaching advocacy skills.

These findings are consistent with an earlier review by Galand and Maticka-Tyndale.^26^

Weak content is compounded by a second problem—weak delivery. The UNESCO/UNFPA review further notes: “Any curriculum rises or falls on the skill of teachers and the culture or environment of the classroom.”^25^ Teachers are often extremely uncomfortable teaching about sexuality and are usually inadequately prepared, as illustrated by a 2006 study in Nepal.^27^ The study reported that “…most of the teachers did not want to deal with sensitive topics and feared censure by their colleagues and society. Some lacked the skills to give such instruction.”^27^ A similar study in 2013, also conducted in Nepal, reiterated that “…students’ needs and expectations regarding HIV and sexual health education are not being met through their schools.”^28^

#### Providing Adolescents With Appropriate SRH Services

A number of evaluations have shown that adolescent use of SRH services can be increased, especially when the following 4 complementary approaches are implemented together^29^^-^^31^:

- Providers are trained and supported to be nonjudgmental and friendly to adolescent clients.
- Health facilities are welcoming and appealing.
- Communication and outreach activities inform adolescents about services and encourage them to make use of services.
- Community members are supportive of the importance of providing health services to adolescents.

While many projects and programs around the world aim to provide “youth-friendly services,” careful examination suggests that most programs do not implement these 4 approaches together. This is illustrated by a study in Brazil, which reported^32^:

The findings indicate that the Project [an integrated school- and health clinic-based adolescent reproductive health initiative] was successful in increasing the flow of sexual and reproductive health information to secondary-school students and that it had an impact on adolescents' intentions to use public health clinics in the future. No effects on sexual or contraceptive-use behaviors or on use of public clinics were observed, however.

The authors admit the project was not well designed^32^:

…although the project trained clinic staff to provide reproductive health services appropriate to adolescents, few of the features of clinics believed to make health services adolescent friendly were incorporated into the project.

Because of practical constraints, the weak design was further compromised by weak implementation, thus it is not surprising that the project did not succeed in increasing adolescent use of clinic services.^32^

An evaluation of the Government of Malawi’s youth-friendly health services (YFHS) program was conducted in 2013.^33^ The YFHS program was initiated in 2007 to make all health services more acceptable, accessible, and affordable to young people, and the evaluation aimed to measure progress since the program was launched. The evaluation found that despite the national attention paid to YFHS, awareness of YFHS was low, with less than one-third of youth surveyed having heard of YFHS and only 13% ever having made use of YFHS. Young people, parents, and community leaders lacked information on and showed only weak support for YFHS, with many adolescents expressing skepticism about the quality of services available. Around 60% of facilities reported having copies of government-issued YFHS standards, but less than one-third reported they were implementing key aspects of the standards including the availability of signage, trained providers, outreach to adolescents, and adolescent-specific educational materials. One of the constraints to providing YFHS, according to some supervisors, was weak supervision. On the plus side, the majority of young people who reported having visited a YFHS facility had done so in the 12 months prior to the assessment and expressed satisfaction with the care received.

### Interventions Are Delivered Piecemeal

Adolescents’ SRH outcomes are determined by a complex web of interrelated factors that operate at different levels.^34^ Individuals make choices to engage in specific behaviors based on what they know, believe, and are able to do. They make these choices within the context of their relationships, families, and communities, their economic circumstances, and the prevailing social norms and traditions. The laws, policies, and regulations may help or hinder their choices, and more often than not, it is the latter. (The reality is that many adolescents have limited control on what they can do and on what happens to them. Especially for girls, their choices are constrained or made by others on their behalf.) In order to improve the health of adolescents, action is needed at each of these levels, often by different sectors other than health.^35^

Yet in many places, interventions are implemented in an uncoordinated and piecemeal fashion, and so—not surprisingly—they do not result in positive outcomes. Sometimes, the fragmented implementation of interventions can even have negative effects. A case in point is a sole focus on legal reform to end harmful traditional practices such as early marriage or female genital mutilation, which can actually drive the practice underground.

A number of advocacy efforts have addressed the need to repeal laws that permit early marriage or to pass new laws that raise the age of marriage and/or prohibit female genital mutilation. A review by Lee-Rife et al. concluded that that there is little evidence that laws on their own make any substantial contribution to discouraging or eradicating child marriage.^19^ In fact, among the 12 African countries where there has been a more than 10% decrease in early marriage, only 3 (Ethiopia, Liberia, and Sierra Leone) have a strong legal framework.^36^ Berg et al. and Johansen et al. similarly conclude that laws alone do not prevent female genital mutilation.^20^^,^^21^ More recently, Mackie argues that an exclusive focus on laws can even lead to greater harm by triggering active legal disobedience.^37^ Efforts to address harmful practices may be more effective if they are linked to holistic interventions, such as keeping girls in schools or strengthening their employment options, and if they address sociocultural norms that support early marriage or female genital mutilation.^38^

There is little evidence that laws on their own make any substantial contribution to ending child marriage or female genital mutilation.

Convincing evidence of the importance of implementing coordinated and complementary interventions comes from a 2014 review of England’s multi-year effort to reduce teenage pregnancies. Beginning in 1999, the Government of the United Kingdom established a 10-year strategy to reduce teenage pregnancy rates. One of the 4 key themes of the strategy was to ensure coordinated action of better prevention activities for boys and girls, which included providing comprehensive sexuality and relationship education through a variety of channels, improving access to contraception through a variety of means, supporting a communication campaign to reach young people and their parents, and strengthening support for young parents.

A midcourse review in 2005 showed that while overall the under-18 conception rate had declined by 11%, there was wide variation in results across other areas. This prompted an in-depth review: 3 local government areas where under-18 conception rates declined since 1998 were compared with 3 areas with similar demographics but where conception rates were static or increasing. The review found that areas with better reduction rates were implementing all aspects of the strategy whereby all relevant agencies were involved to create a “whole systems” approach. Strong leadership was also an important factor. In areas with little progress, only some aspects of the strategy were being implemented, even though considerable effort was being made. As a result of the 2005 review, the Government identified and disseminated nationwide 10 “must-do” activities, and it established a system for self-assessment and external assessment. Teenage pregnancy rates began to decline in all 150 local government areas of the country, and the decline continues to this day.^39^

### Interventions Are Delivered in a Low “Dosage” and Are Not Sustained

Dosage refers to how intensively and/or how long an intervention or a package of interventions is delivered.^40^ In practical terms this means a program that reaches young people with complementary messages using a variety of delivery mechanisms (e.g., teaching sessions in school, billboards, and radio or television chat shows), has a higher “dosage” than another program that uses fewer and less-intensive approaches. An outreach worker who conducts monthly discussion sessions with a youth group over a period of 12 months also delivers an intervention with “higher dosage” than another who only conducts a single session.

Dosage matters. A review of behavioral interventions to reduce HIV, sexually transmitted infections, and pregnancy in adolescents showed that programs delivered with greater intensity or for a longer duration were more effective than shorter programs,^41^ perhaps because they allow for more in-depth discussion of and reflection on cultural and gender norms and other social structures that have a powerful effect on individual behaviors and capacity to change.

Behavioral interventions delivered with greater intensity or for longer duration are more effective than shorter programs.

For programs to improve and change knowledge, understanding, attitudes, beliefs, and behaviors over the long term and at the community level, programs must be delivered regularly, consistently, and with intensity over a sustained period of time. We do not yet know what level of intensity and/or duration is optimal. What we do know is that interventions (or dosages) that are not sustained do not bring about long-term change at the community level. A striking example of this comes from a 20-month comprehensive community-based sex education and reproductive health service project in Shanghai, China. At the end of the project, the endline survey indicated the project had had a positive effect on contraceptive use among unmarried youth who had been exposed to the intervention.^42^ A little over 2 years (28 months) after the end of the project, a follow-up survey found that without the consistent and sustained dosage from the intervention, young people appeared to revert to behaviors seen at baseline.^43^

## NEW FRONTIERS

There is a strong need for coordinated and complementary approaches that improve the health of adolescents as well as a need to abandon those approaches that are wasteful and ineffective. Furthermore, we need to explore new approaches that show promise, such as strengthening private-sector engagement to reach adolescents and improving adolescent access to long-acting reversible contraceptives (LARCs).

According to Catalano et al.,^44^ the standard approaches to improving adolescent health have focused on health promotion, prevention, and treatment of “problem behaviors” often with a focus on a single behavior, such as abstinence, delay of sexual initiation, or contraceptive/condom use. These single-focus prevention interventions have been criticized, and greater consideration of the co-occurrence of problem behaviors and improved understanding of the overlap in predictors across many behaviors is needed.^44^

In a growing number of high-income countries, the adolescent health field is embracing the concept of prevention science. The Society for Prevention Research indicates the primary goal of prevention science is “to improve public health by identifying malleable risk and protective factors, assessing the efficacy and effectiveness of preventive interventions and identifying optimal means for dissemination and diffusion.”^45^ Catalano et al. note that behavioral factors are the major cause of adolescent morbidity and mortality.^44^ Based on a number of controlled trials, they advocate for the implementation of interventions that simultaneously address risk factors and also increase those protective factors that mitigate against risky behaviors. The research base that has been developed in high-income countries has recently begun to be applied to low- and middle-income countries by translating existing approaches and developing and testing new preventive interventions in lower-income contexts.^44^

## CONCLUSION

Research studies and programmatic experiences over the last 20 years have without a doubt improved our knowledge and understanding of adolescent programs and interventions. We have a clearer understanding of the effective components of sexual and reproductive health education and health services. We are also better at delivering programs to adolescents, with greater consideration of the antecedents that influence the sexual and reproductive health of adolescents.

Nevertheless, there are still glaring gaps in our knowledge and understanding of effective adolescent health programming, especially at scale. With a sizeable and growing youth population, it is urgent that we accelerate the expansion of proven approaches while safeguarding fidelity to those factors that ensure quality and success. In any event, we must stop the implementation of ineffective interventions that waste human and financial resources and raise questions about the value of policies and programs that do not demonstrate results. Further, we recommend greater attention to the adaptation of evidence-based prevention science approaches that simultaneously address risk and protective factors for adolescents in lower- and middle-income countries. This should include the creation of a database that documents best and promising practices in prevention science and adolescent health.

We must stop implementing ineffective interventions that waste human and financial resources.

## Notes

**Peer Reviewed**
